# Twist of Fate: A Case Series on Intestinal Malrotation in Adult Patients

**DOI:** 10.7759/cureus.72763

**Published:** 2024-10-31

**Authors:** Amol Wagh, Najmeh Mirkhushal, Thrilok Gowda, Abhipray P Ramteke, Manikantha G

**Affiliations:** 1 General Surgery, Grant Government Medical College and Sir JJ Group of Hospitals, Mumbai, IND

**Keywords:** acute abdominal pain, intestine, ladd’s band, midgut malrotation, volvulus of midgut

## Abstract

Intestinal malrotation is a congenital anomaly characterized by rotational errors during the embryonic development of the midgut. Although it is mainly a pediatric diagnosis, some cases do present in adult life. In adults, the condition can present in various ways, with symptoms that may be acute, acute on chronic, chronic, or discovered incidentally. This case series highlights the diverse presentations and management of midgut malrotation in adult patients.

## Introduction

Midgut malrotation is a developmental rotational anomaly of the embryonic bowel. Malrotation can present in different ways: acutely with signs of obstruction, intermittently typically arising due to partial or intermittent obstruction of the intestines or asymptomatically.. The diagnosis of malrotation of the intestine represents acute surgical emergencies like intestinal volvulus [1, 2].

The incidence of rotational anomalies of the midgut is difficult to determine but is estimated to be 1 in 6000 live births [3]. Intestinal malrotation presenting in an adult is a very rare entity. It accounts for 0.2% to 0.5% of all cases of intestinal malrotation [4]. Most of these cases are asymptomatic and diagnosed incidentally on radiological imaging or unrelated surgery [2].

The midgut normally herniates out of the coelomic cavity through the umbilical ring at approximately the fourth week of fetal development. By the tenth week of gestation, the intestine begins to migrate back into the abdominal cavity in a counterclockwise rotation around the axis of the superior mesenteric artery (SMA) for 270 degrees. The duodenojejunal segment returns first, rotating beneath and to the right of the superior mesenteric artery (SMA) to settle in the left upper quadrant at the ligament of Treitz. Similarly, the cecocolic segment rotates counterclockwise around the SMA, positioning itself in the right lower quadrant. By week 12, this process of intestinal rotation is complete, and the colon becomes fixed to the retroperitoneum. An interruption or reversal of any of these coordinated movements implies an embryologic explanation for the range of anomalies seen.

Midgut malrotation can occur in various forms. The first type is non-rotation, in which the small bowel stops rotating at 90 degrees counterclockwise and is the most common form. Another form is incomplete rotation, in which the duodenum or right colon partially rotates. A rarer type is reverse rotation, in which the midgut rotates clockwise [5].

## Case presentation

Case 1

A 14-year-old female with no comorbidities was admitted to the emergency ward with acute-onset abdominal pain and multiple episodes of bilious vomiting for 3 days. The patient did not experience fever, altered bowel habits, or malena. She did not give any history of previous surgeries. She was hemodynamically stable. Physical examination revealed a soft abdomen with no guarding, tenderness, or rigidity. Initial biochemical tests show white cell counts of 15,000 cells per microliter, lactate level of 2 mmol/L, and C-reactive protein(CRP) level of 0.9 mg/dL. She was started on intravenous fluids, proton pump inhibitors, antiemetics, and continuous nasogastric tube aspiration. Ultrasonography of the abdomen reveals a reverse superior mesenteric artery(SMA)-superior mesenteric vein(SMV) relationship, which was suggestive of intestinal malrotation.

**Figure 1:**
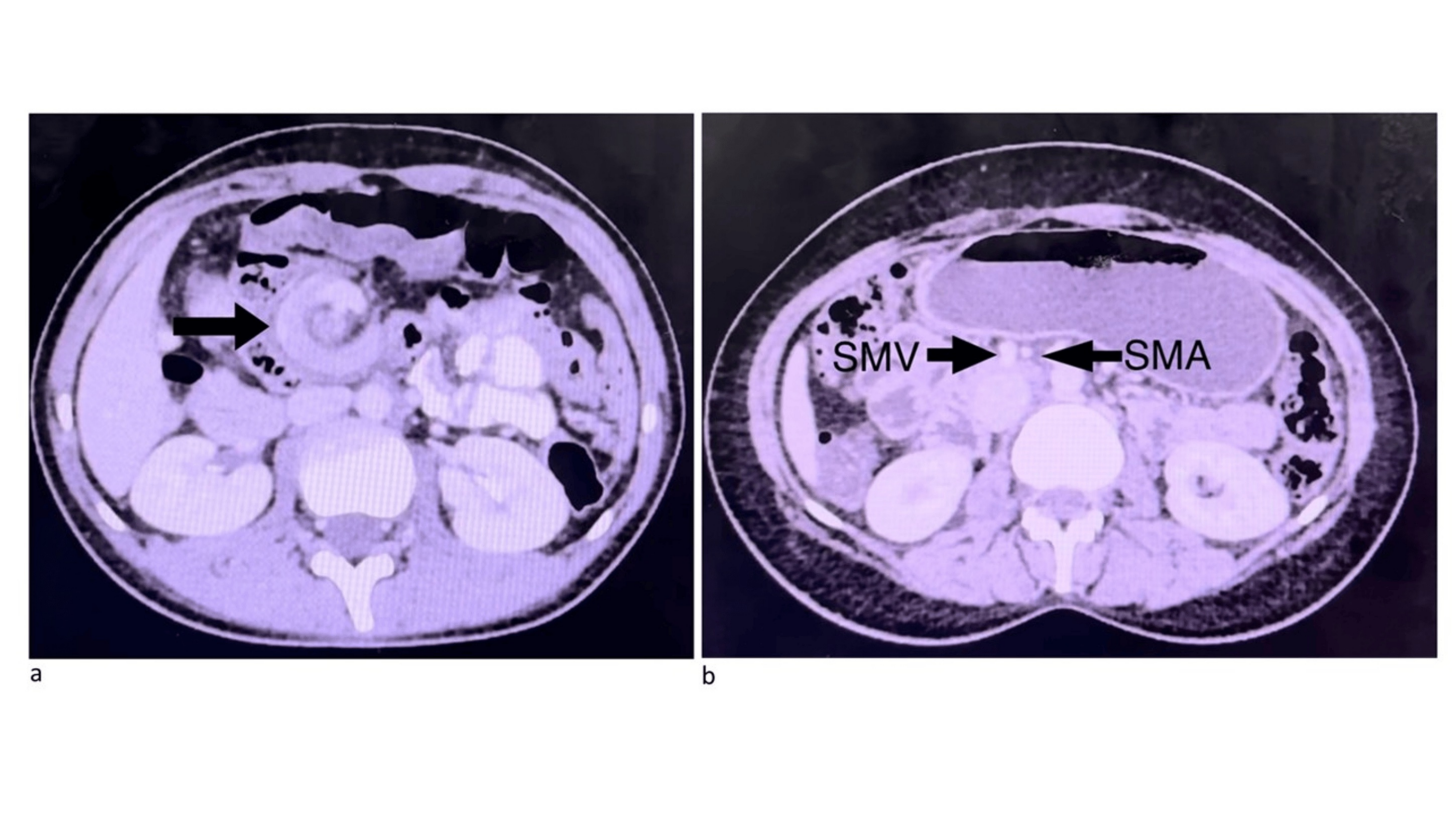
a) Axial view CECT showing classical whirlpool sign; b) Axial view CECT showing inverse relationship of SMV and SMA CECT: contrast-enhanced computed tomography, SMV: superior mesenteric vein, SMA: superior mesenteric artery

Contrast-enhanced computed tomography (CECT) of the abdomen revealed twisting of the duodenum and proximal jejunum along with its mesentery around the SMA with twisting of SMA and SMV (Figure 1).

**Figure 2:**
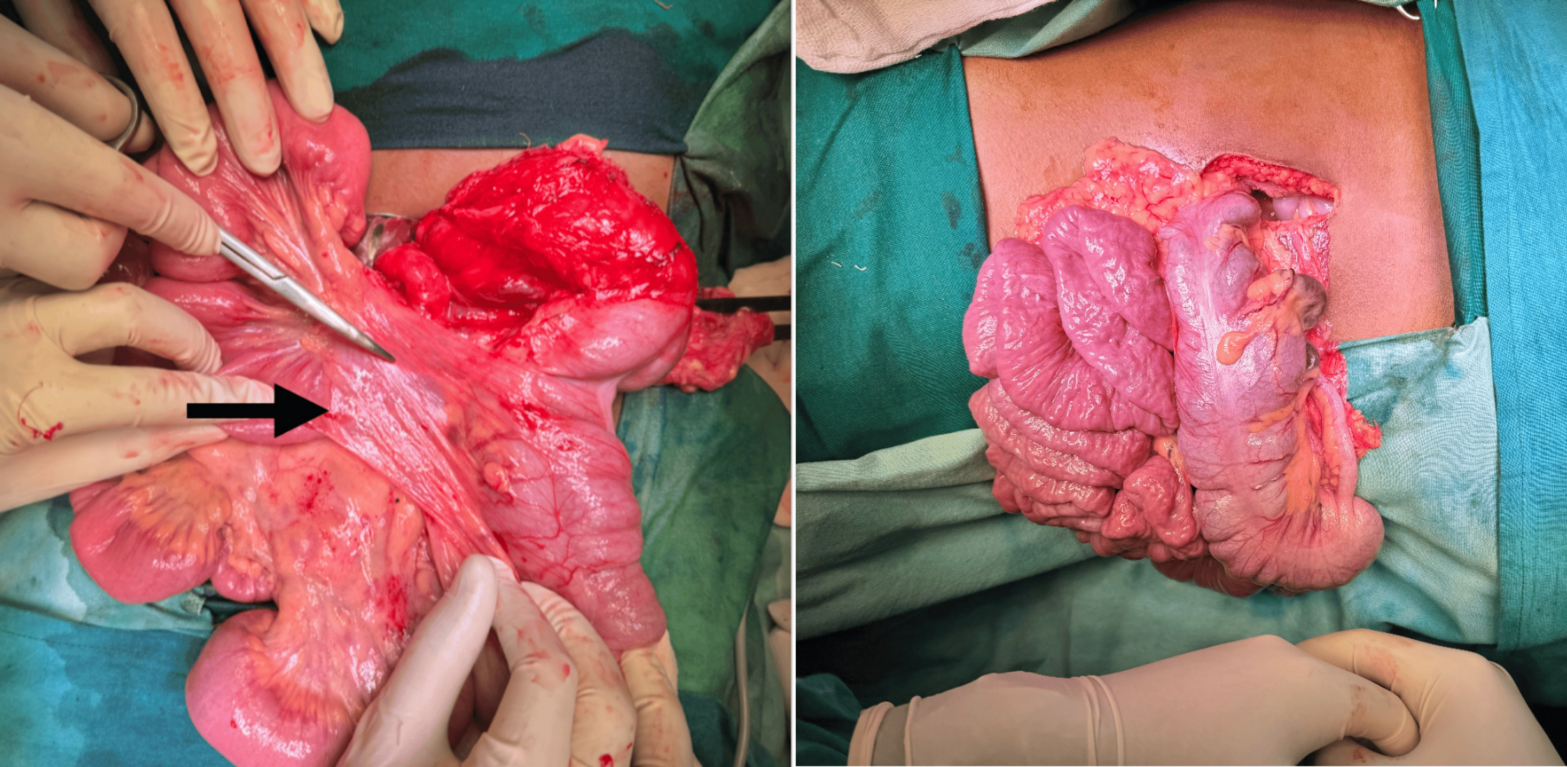
Intraoperative images showing Ladd's band and large and small bowel not attached to posterior wall with mesentery.

Given the radiological findings, the patient underwent emergency exploratory laparotomy due to volvulus. Intra-operatively, duodenojejunal (DJ) flexure was seen arising from the left side of the bowel. SMV appeared to be dilated. Cecum was present in the midline (Figure 2). Ladd’s procedure was done in which the duodenum was de-rotated in the anticlockwise direction, and the bands identified between the duodenum and the vascular pedicle were then released. The appendix appeared normal and was ligated and removed. The rest of the small and large bowel was well perfused and, hence, did not require any further resection. A large bowel was kept on the left side of the abdomen. The small bowel was kept on the right side of the abdomen.

She made a quick recovery post-operatively. Her hospital stay was unremarkable, and she was discharged on the fifth postoperative day. She was symptom-free on her last follow-up 1 month after the surgery.

Case 2

A 36-year-old female with no comorbidities presented with recurrent pain in the abdomen for one and a half years with a history of multiple episodes of non-bilious vomiting. The patient did not give a history of yellowish discoloration of skin, sclera, or fever. She did not give any history of previous surgeries. She has uterine agenesis. Physical examination revealed a soft abdomen with no guarding, tenderness, or rigidity. Ultrasonography of the abdomen revealed cholelithiasis adenomyomatosis with changes of chronic cholecystitis, ectopic right kidney and absent uterus. Laboratory tests showed white cell counts of 7,000 cells/μL (reference range: 4,500-11,000 cells/μL) and a CRP level of 0.2 mg/dL (reference range: 0.8-1.0 mg/dL).

Further, CECT revealed duodenal non-rotation. The ﻿D3 segment of the duodenum does not cross the midline and lies on the right side of the SMA and the aorta. The cecum and Ileocecal (IC) junction are seen in the midline of the pelvis. 

Midgut malrotation was incidentally found, and the patient was also not symptomatic, so she was not intervened for the same. The patient underwent laparoscopic cholecystectomy. Intraoperatively, the cecum and IC junctions are seen in the midline of the pelvis. No Ladd's bands were observed. The appendix was seen arising from the IC junction in the midline. Sigmoid colon was in the right iliac fossa.

Case 3

A 19-year-old male presented with chief complaints of generalized worsening abdominal pain and non-bilious vomiting for 4 days. The patient has no co-morbidities and no previous surgical history. On abdominal examination, there was no tenderness or guarding. Laboratory tests revealed white cell counts of 6,000 cells per microliter and a lactate level of 1 mmol/L.

**Figure 3:**
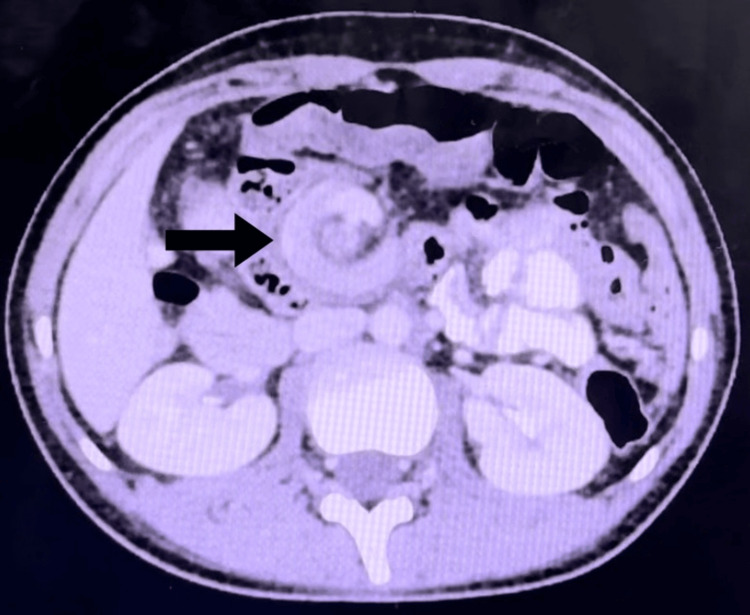
CECT axial view showing that the relationship between the SMA and SMV is inverted. CECT: contrast-enhanced computed tomography, SMV: superior mesenteric vein, SMA: superior mesenteric artery.

CECT abdomen revealed ﻿DJ flexure is whirling around the superior mesenteric vessels, giving the whirlpool sign, which suggests intestinal malrotation (Figure 3).

The patient was managed conservatively as there was no volvulus of the intestine. On day two of admission, the patient passed flatus, following which oral sips followed by clear liquids started. On day three, the patient passed stools. The patient was then discharged with a plan to follow up after 1 month, during which time she continued to remain asymptomatic.

Case 4

**Figure 4:**
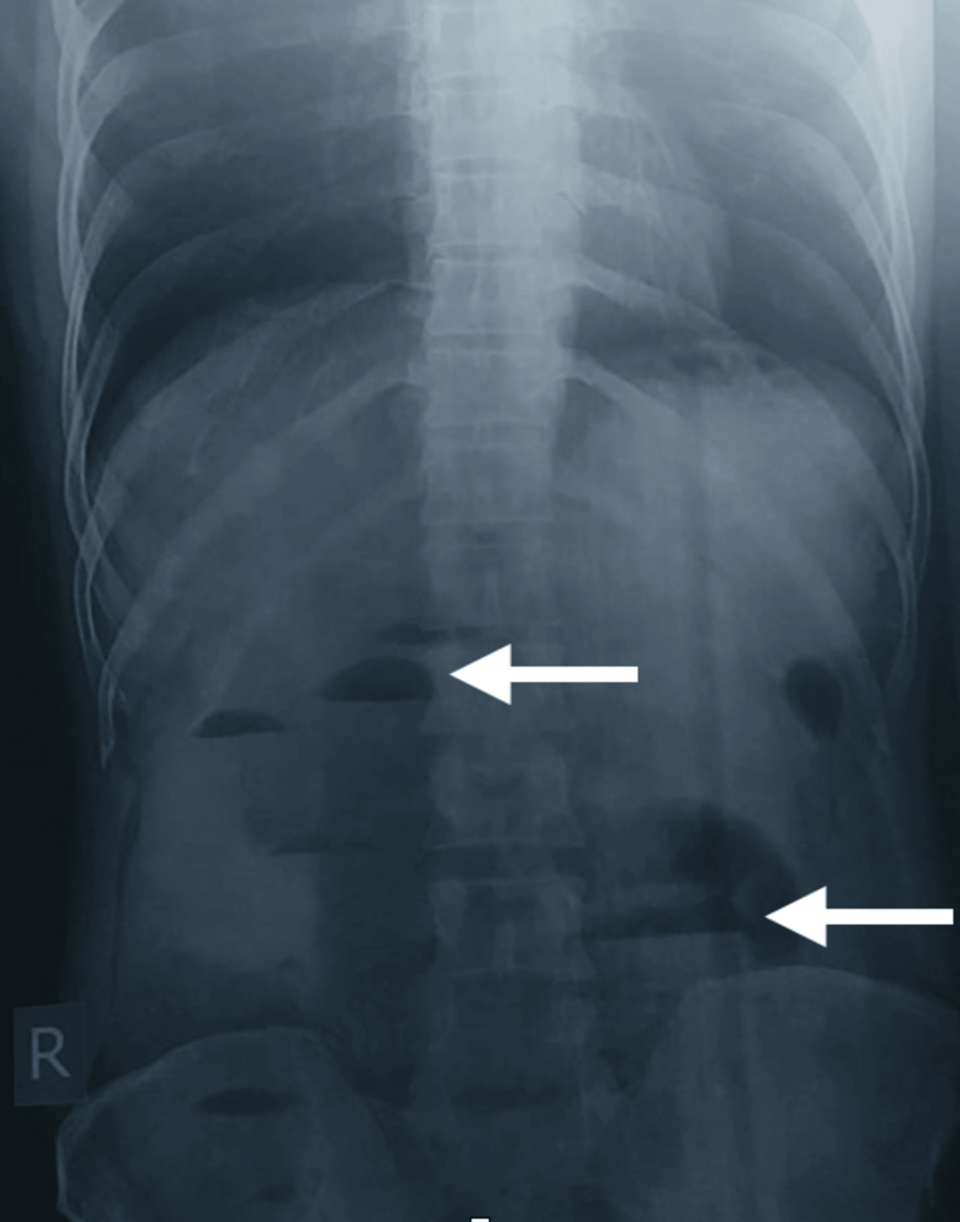
X-ray abdomen erect showing multiple air fluid levels.

A 26-year-old male presented with chief complaints of pain in the abdomen for three days, which was worsening despite taking analgesics. Laboratory tests revealed white cell counts of 18,000 cells per microliter, a lactate level of 3 mmol/L, and a creatinine level of 2.1 mg/dL. On physical examination of the abdomen, guarding was present. On radiological investigation, an abdominal erect X-ray revealed multiple air-fluid levels (Figure 4). CECT was not done due to raised creatinine levels.

**Figure 5:**
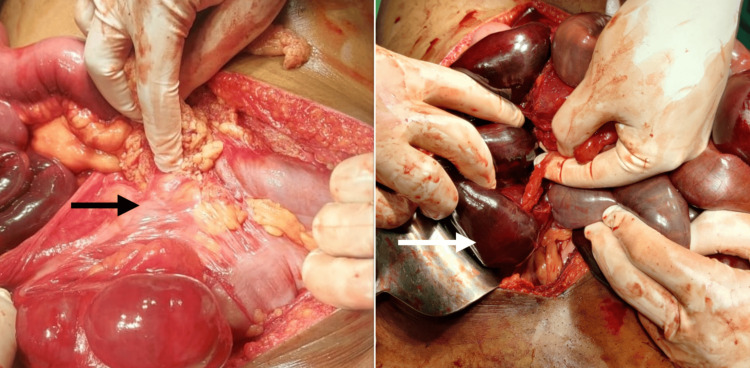
Intraoperative images showing Ladd’s band and gangrenous midgut (jejunum and ileum) of about 140cm.

The patient underwent an emergency exploratory laparotomy. Intraoperatively, he had a right paraduodenal hernia with midgut incomplete rotation; the small bowel was on the right side, and the cecum and ascending colon were on the left side. Midgut was gangrenous. Around 140 cm of small bowel was resected, a stoma was created, and Ladd's bands were released (Figure 5).

He made a quick recovery post-operatively with optimal stoma output. He was symptom-free on her last follow-up one month after the surgery.

## Discussion

Intestinal malrotation is a congenital condition caused by the incomplete or abnormal rotation of the bowel during embryonic development. First identified by Franklin Mall in 1897, the formation and positioning of the human intestine follow a precise sequence of stages: herniation, rotation, retraction, and fixation [1]. Any disruption in this process results in malrotation, leading to a shortened mesenteric root, significantly heightening the risk of volvulus.

Stringer classified malrotation into three primary types: Type I (non-rotation), where the duodenojejunal (DJ) junction is on the right and the colon on the left; Type II (duodenal malrotation), with the cecum located in the epigastric region overlying the third part of the duodenum; and Type III (combined duodenal and cecal malrotation), where the DJ loop is positioned anterior to the superior mesenteric artery (SMA) and the transverse colon is located posterior to the SMA [5].

An acute presentation is more commonly associated with incomplete rotation, typically appearing in infancy with symptoms of obstruction, such as bilious vomiting and distension, which may progress to peritonitis. Hematochezia, indicating mucosal sloughing, is a sign of poor prognosis. A chronic presentation is more frequent after five years of age and may be asymptomatic or involve intermittent abdominal pain lasting 45-60 minutes that resolves spontaneously. However, acute abdomen can occur at any age [6].

Diagnosis of malrotation and volvulus

As most children present with obstruction, the diagnosis is fairly straightforward. It presents more of a challenge in adults who do not present with acute obstruction. A high index of suspicion is needed in adults with recurrent, non-specific abdominal pain, especially with left-sided pain where diverticulitis is considered the primary differential. A plain radiograph is almost always the first investigation that can show features of obstruction. Ultrasonography of the abdomen is highly sensitive and specific for volvulus. SMV is seen lying to the left or anterior to SMV, with vessels twisting around the base of the mesentery. CECT of the abdomen provides a definitive diagnosis for the type of malrotation, with or without volvulus. Routine screening is not recommended, except in infants with heterotaxy.

Management

The treatment for malrotation presenting with obstruction or volvulus (symptomatic/asymptomatic) is surgical intervention. In cases of volvulus, the procedure involves exteriorizing the bowel and performing a de-rotation. If the bowel viability is questionable, the patient must be taken up for a second look laparotomy after 24 hours [7]. Once bowel viability is established and de-torsion is finished, Ladd's procedure is performed. After this, any extrinsic compression is checked for around the duodenum, cecum, and colon. The transverse colon is assessed carefully for reverse rotation/second volvulus. An appendectomy is done because, in the future, it may present a diagnostic dilemma if the patient suffers appendicitis and the appendicular artery may be unknowingly damaged during the dissection of Ladd’s bands.

Ladd’s Procedure

It is performed only after dealing with volvulus. The procedure consists of four steps: adhesiolysis of bands between the colon and lateral abdominal wall to straighten the duodenum and relieve obstruction; adhesiolysis of bands over the mesentery to widen its base and prevent further obstruction; appendectomy using the inversion ligation technique to maintain a clean-contaminated procedure; placement of the cecum in the left lower quadrant to maximize mesenteric broadening [8].

In our cases 1 and 4, patients presented with features of obstruction, prompting immediate surgical intervention. Immediate surgical management is usually not required for incidentally detected malrotation findings on radiological investigations.

## Conclusions

This case series highlights the diverse presentations and management of midgut malrotation in adult patients. The clinical manifestations ranged from acute abdominal pain and bilious vomiting to chronic recurrent abdominal pain and incidental findings during imaging for unrelated conditions. The severity of symptoms varied, with some patients requiring urgent surgical intervention, such as the Ladd's procedure or bowel resection, while others were managed conservatively due to the absence of acute symptoms.

In conclusion, midgut malrotation can present a wide spectrum of clinical scenarios. A high index of suspicion, with radiological investigations, is essential for diagnosis. Surgical intervention, when indicated, is effective in alleviating symptoms and preventing life-threatening complications such as bowel ischemia and gangrene. Regular follow-up is recommended to ensure long-term well-being and to monitor for any recurrence of symptoms.
